# Fermented foods and probiotic consumption frequency as protective indicators for peri-implant diseases – a cross-sectional study

**DOI:** 10.1186/s12903-024-04625-8

**Published:** 2024-07-26

**Authors:** Tugba Sahin

**Affiliations:** Division of Periodontology, Faculty of Dentistry, Abant İzzet Baysal University, Bolu, Turkey

**Keywords:** Dental implants, Diet, Fermented foods, Probiotics, Peri-implantitis

## Abstract

**Background:**

Due to their modulatory effect on biofilm growth, bacterial gene expressions, and host-modulation effects, fermented foods and probiotic products could potentially have a protective role against peri-implant diseases. This cross-sectional study aimed to examine the association of consumption of fermented foods and products containing probiotics, with peri-implant health and diseases.

**Methods:**

A total of 126 implants were included. The peri-implant health status (peri-implantitis, peri-implant mucositis, and peri-implant health) was assessed through Chicago’s Classification of periodontal and peri-implant Diseases and Conditions. A questionnaire was used to evaluate the consumption patterns of fermented and probiotic foods and product. One-way ANOVA was employed to compare the 3 peri-implant conditions categories in terms of fermented food and probiotic consumption.

**Results:**

There were significant differences in the daily and general consumption of yogurt, probiotic yogurt, kefir, ayran, vinegar, pomegranate syrup, whole meal bread, and homemade butter among peri-implantitis, peri-implant mucositis and peri-implant health (*p* < 0.05). The peri-implant health group consumed significantly more yogurt, kefir, ayran, vinegar, whole wheat bread, and homemade butter than peri-implant mucositis and peri-implantitis.

**Conclusion:**

A higher consumption of fermented and probiotic foods may be associated with peri-implant health. Fermented and probiotic products may be useful for prevention of peri-implant diseases in patients with implants.

**Supplementary Information:**

The online version contains supplementary material available at 10.1186/s12903-024-04625-8.

## Background

The successful use of osseointegrated dental implants has dramatically changed dentistry [1]. However, peri-implantitis is a common complication associated with dental implants in the long term [2–5]. Given the limited efficacy of its current treatment approaches [6, 7], the modern management of peri-implantitis is based on its prevention and early diagnosis [8, 9]. Ozone, glycine/erythritol, probiotics, hydrogel, and chlorhexidine gel can be used in addition to mechanical debridement in non-surgical peri-implant treatment [10, 11].

In prevention, the main strategies are the treatment of peri-implant mucositis as a precursor of peri-implantitis and interventions aimed at targeting modifiable risk/protective factors [12, 13]. Specifically, peri-implantitis is considered an inflammatory microbially-driven disease [14–17]. Compared to the periodontal microbiota, the peri-implant microbiota represents a bacterial ecosystem that shows significant diversity among patient, with some bacterial genera being quantitatively superior. In fact, many studies illustrated the modification of submucosal microbiota following the transition from peri-implant health to peri-implant disease [18]. Therefore, strategies aiming at modulating the composition of the peri-implant microbiota or at favoring an effective host-response have received attention as possible preventive measures.

Certain fermented foods belong to a subgroup of functional foods known as probiotics, as they contribute to promoting health [19]. Bacteriocins such as *Lactobacillus plantarum* D4 act against pathogens in fermented foods and may exhibit antibacterial activities [20]. In addition, the fermentation of foods reduces the risk of contamination by pathogenic microorganisms through the production of antibacterial metabolites such as organic acids and ethanol [21]. Probiotic bacteria are effective owing to their antagonistic effects against certain bacteria, antimicrobial agent release against pathogenic bacteria, and the competitive exclusion principle [22]. Based on these effects, fermented foods and probiotics could potentially have a protective role against peri-implant diseases, as they have shown for periodontitis [23, 24]. This study aimed to determine the relationship between the frequency of fermented food consumption and use of probiotic products with peri-implant health and diseases.

## Methods

### Study settings

The research was conducted at a single institution, namely, the Division of Periodontology within the Bolu Abant İzzet Baysal University Faculty of Dentistry. Participants were informed verbally and in writing about the design of the study. The study was conducted with respect to Helsinki Declaration. Informed consent form was obtained from all participants.

### Sample size calculation

To assess the statistical significance of the disparities among the groups categorized as peri-implant health, peri-implant mucositis, and peri-implantitis, a one-way analysis of variance (ANOVA) was planned. To highlight an effect size of 0.35, with power of 85% and an alpha-error level of 5%, each group should have consisted of at least 42 implants.

### Selection of participants

Accordingly, this study included 126 subjects with treated periodontitis, each one contributing with one implant. The inclusion criteria for patient involvement included age between 18 and 70 years, with a minimum of 20 natural teeth within the oral cavity, having overall systemic health, and being no-smokers. Systemically unhealthy patients who did not use antibiotics within the past month, were breastfeeding or pregnant, had uncontrolled diabetes, rheumatic fever, a history of lung or kidney disorders, and used drugs that could affect periodontal tissues were not included in the study. Study participants were also selected based on their nonparticipation in a specific dietary regimen.

Potential confounding variables such as smoking habits, systemic diseases, regular medication use, antibiotic intake, and adherence to specific dietary regimens prescribed by a dietitian or to a self-administered regimen were excluded. Excluding these variables allowed for a more accurate interpretation of the findings.

### Peri-implant health status

Peri-implant health status was assessed by one clinician (T.Ş.) applying the 2017 World Workshop on Classifying Periodontal and Peri-Implant Diseases and Conditions and the 2023 European Federation of Periodontology’s Guidelines [25, 26]. After the examination, the patients were divided into three groups according to their disease status: peri-implantitis (42 implants), peri-implant mucositis (42 patients), and peri-implant health (42 patients).

### Questionnaire

The demographic characteristics (age, weight, sex, height, education, and employment status) of the participants were collected using a questionnaire. In this survey, fermented foods and probiotic products such as bacon, soy sauce, pickles, sourdough bread, whole grain, rye, whole meal bread, ayran, kefir, vinegar (homemade), pomegranate syrup (homemade), probiotic beverages, frequency and amount of consumption of cheese, dark chocolate, tablets, capsules, sachets, butter (homemade), and others (including kimchi, sauerkraut, miso soup, fermented herring, kombucha) were also determined. Data on the individual frequency and consumption of fermented food and probiotic products were recorded, taking into account not only specific but also a wide range of traditional and universal foods available in the market and restaurants. Foods were evaluated using a 7-point Likert-type frequency form (every day, 1–2 per week, 3–4 per week, 1 per month, 2 per month, 1 per year, never). Additionally, the amount of food consumed each time was recorded, and the daily consumption amounts were obtained by dividing them by the frequency of consumption. The questionnaire examining the frequency of consumption of fermented foods and probiotic products was designed according to Turkish dietary guidelines [27]. The questionnaire used in this study, the Food Consumption Frequency Questionnaire, is a validated tool known for its reliability and validity in assessing food frequency consumption. Previous studies [28, 29] have confirmed its accuracy and relevance. This instrument was selected due to its comprehensive coverage of aspects relevant to our research objectives. Its established reliability and validity ensure consistent and accurate data collection. Additionally, it includes measures pertinent to the study population and questions, making it a suitable choice for the study’s goals.

### Statistical analyses

This study utilized the SPSS 26.0 software for data analysis. Descriptive characteristics regarding all the covariates were initially summarized. One-way ANOVA was employed to compare the study groups in terms of fermentable food and probiotic consumption. Statistical significance was set at *p* < 0.05.

## Results

Among the participants included in this study, 56.7% were males, and 43.3% females. Table 1 presents the participants’ general characteristics, which were similar among the three groups.

**Table 1:** Age, weight and height information of participants

	Min	Maks	Mean values	S.D.
Weight	49	98	75,5	11,4
Height	155	185	166,8	9,9
Age	27	70	52,5	10,5

Table 2 shows the total food consumption among the groups. Significant differences were observed in terms of consumption amount in probiotic yogurt, whole meal bread, and kefir. Specifically, probiotic yogurt consumption was higher in the patients with peri-implant mucositis. Individuals with healthy peri-implant tissues consumed more whole meal bread and kefirs.

**Table 2:** Comparison of participants’ groups by food consumption amount

	Peri-implantitis	Peri-implant mucositis	Peri-implant health	General	*p*
Yogurt	212,5 ± 99,2	197,5 ± 11,2	187,5 ± 50,0	200,8 ± 68,0	0,512
Boza	8,3 ± 40,8	50,0 ± 88,9	50,0 ± 89,4	33,3 ± 75,2	0,008*
Probiotic yogurt	16,7 ± 56,5	47,5 ± 85,0	25,0 ± 68,3	29,2 ± 70,3	0,043*
Tarhana	191,7 ± 40,8	192,5 ± 33,5	200,0 ± 73,0	194,2 ± 48,8	0,858
Sausage	32,1 ± 33,2	43,5 ± 50,9	42,2 ± 30,5	38,6 ± 39,1	0,581
Bacon	3,8 ± 7,7	20,0 ± 55,2	11,9 ± 27,1	11,3 ± 35,2	0,317
Soy Sauce	0,0 ± 0,0	3,0 ± 11,3	8,1 ± 25,1	3,2 ± 14,6	0,226
Pickle	80,4 ± 76,5	102,8 ± 218,9	80,3 ± 104,3	88,0 ± 144,4	0,858
Sourdough bread	24,0 ± 26,0	40,0 ± 31,8	32,8 ± 29,9	31,7 ± 29,4	0,196
Whole grain bread	16,7 ± 22,9	22,5 ± 25,5	31,3 ± 31,0	22,5 ± 26,3	0,232
Rye bread	9,4 ± 20,6	16,3 ± 27,2	28,1 ± 74,7	16,7 ± 43,3	0,414
Wholemeal bread	14,6 ± 29,4	25,0 ± 48,0	82,8 ± 158,3	36,3 ± 90,9	0,015*
Ayran	188,8 ± 63,9	229,0 ± 65,9	193,8 ± 106,3	203,5 ± 78,7	0,205
Kefir	63,8 ± 120,0	89,0 ± 132,4	150,0 ± 154,9	95,2 ± 136,4	0,014*
Probiotic beverage	8,3 ± 40,8	20,0 ± 61,6	14,4 ± 50,1	13,8 ± 50,3	0,751
Probiotic cheese	5,0 ± 16,9	12,0 ± 28,2	5,6 ± 16,3	7,5 ± 21,1	0,512
Probiotic dark chocolate	5,0 ± 16,9	13,0 ± 31,3	17,5 ± 31,5	11,0 ± 26,6	0,324
Butter (natural-homemade)	137,5 ± 140,7	163,0 ± 146,2	156,9 ± 124,2	151,2 ± 136,6	0,816
Probiotic tablet, sachet	0,0 ± 0,0	1,5 ± 6,7	0,6 ± 2,5	0,7 ± 4,1	0,482
Pomegranate syrup	12,5 ± 40,7	15,0 ± 43,9	10,6 ± 5,7	12,8 ± 35,8	0,936
Vinegar (Homemade)	17,7 ± 46,3	25,5 ± 60,0	8,1 ± 4,0	17,8 ± 45,2	0,526
Others	0,0 ± 0,0	0,0 ± 0,0	0,0 ± 0,0	0,0 ± 0,0	-

The daily intake of yogurt, probiotic yogurt, whole wheat bread, ayran, kefir, and butter (homemade) also varied among the groups (Table 3). Specifically, the peri-implant health group consumed higher quantities of yogurt, ayran, whole wheat bread, kefir, and butter (homemade) daily. The peri-implant mucositis group consumed more probiotic yogurt daily.

**Table 3:** Comparison of participants’ groups according to daily intake

	Peri-implantitis	Peri-implant mucositis	Peri-implant health	General	*p*
Yogurt	106,9 ± 77,5	101,0 ± 72,1	165,0 ± 66,3	120,5 ± 76,6	0,021*
Probiotic yogurt	0,3 ± 1,3	11,0 ± 44,5	0,9 ± 3,3	4,0 ± 25,8	0,038*
Boza	0,0 ± 0,1	0,4 ± 1,5	8,8 ± 26,3	2,5 ± 13,8	0,001*
Tarhana	46,4 ± 45,1	56,6 ± 56,9	77,5 ± 77,5	58,1 ± 59,3	0,269
Sousage	4,9 ± 6,5	6,2 ± 8,2	6,5 ± 10,0	5,8 ± 8,0	0,785
Bacon	2,4 ± 8,3	6,5 ± 27,9	4,4 ± 13,1	4,3 ± 18,0	0,763
Soy souce	0,0 ± 0,0	2,5 ± 11,2	0,7 ± 2,5	1,0 ± 6,6	0,441
Pickle	24,4 ± 27,3	28,2 ± 46,1	37,5 ± 61,8	29,2 ± 44,4	0,66
Soudough bread	13,1 ± 16,9	28,6 ± 30,2	19,4 ± 20,0	19,9 ± 23,5	0,091
Whole grain bread	8,8 ± 14,9	18,3 ± 24,0	21,3 ± 31,4	15,3 ± 23,5	0,203
Rye bread	6,8 ± 18,4	9,4 ± 20,2	5,5 ± 13,5	7,3 ± 17,7	0,802
Wholemeal bread	10,8 ± 25,6	15,2 ± 28,5	33,0 ± 63,7	18,2 ± 40,4	0,019*
Ayran	67,9 ± 69,4	92,0 ± 60,7	105,4 ± 83,8	86,0 ± 71,4	0,042*
Kefir	10,4 ± 40,8	25,3 ± 61,0	70,0 ± 91,4	31,2 ± 67,6	0,019*
Probiotic beverage	1,7 ± 8,2	0,0 ± 0,1	1,9 ± 7,5	1,2 ± 6,4	0,617
Probiotic cheese	3,8 ± 13,5	4,7 ± 12,2	2,1 ± 7,5	3,6 ± 11,6	0,811
Probiotic dark chocolate	1,0 ± 3,4	5,5 ± 15,0	7,5 ± 16,5	4,2 ± 12,4	0,232
Butter (homemade)	83,7 ± 127,9	144,8 ± 145,8	156,3 ± 125,0	123,1 ± 135,0	0,017*
Probiotic tablet, sachet	0,0 ± 0,0	1,5 ± 6,7	0,6 ± 2,5	0,7 ± 4,1	0,482
Pomegranate syrup	11,2 ± 41,0	3,2 ± 5,4	7,3 ± 4,3	7,5 ± 26,1	0,610
Vinegar (Homemade)	9,5 ± 23,2	12,9 ± 44,2	6,9 ± 4,7	10,0 ± 29,1	0,829
Others	0,0 ± 0,0	0,0 ± 0,0	0,0 ± 0,0	0,0 ± 0,0	-

There was a statistically significant difference also in consumption frequencies of yogurt, kefir, pomegranate syrup, vinegar, and probiotic tablets between the groups. Specifically, the peri-implant health group had higher daily consumption frequencies of yogurt, kefir, and pomegranate syrup than the other groups. Unlike the other groups, the peri-implantitis group did not consume probiotic tablets. Additionally, the proportion of those who stated that they did not consume any of these foods was lower in the peri-implant health group than in the other groups (Supplementary Table 1).

There is a statistically significant negative correlation between kefir consumption and probing depth (r: -0.309, *p* < 0.05), bleeding on probing (r: -0.268, *p* < 0.05), and clinical attachment level (r: -0.320, *p* < 0.05). The occurrence of these three parameters decreased as kefir consumption increased. There is a statistically significant negative and low-level strong relationship between whole grain bread consumption and plaque index (r: -0.321, *p* < 0.05). The plaque index value decreased as whole-grain bread consumption increased (Table 4).

**Table 4:** Comparison of the relationship between periodontal parameters and demographic characteristics and consumption of fermented foods and probiotic products

	Plaque Index	Gingival Index	Probing Depth	Bleeding on Probing	Gingival Recession	Clinical Attachment Level
Yogurt	-0.043	-0.041	0.125	0.008	-0.078	0.118
Probiotic yoghurt	0.053	-0.048	0.044	0.131	0.117	0.040
Boza	0.046	0.035	-0.048	-0.046	0.121	-0.053
Tarhana	0.191	0.058	0.049	-0.034	0.034	0.051
Sausage	0.055	0.217	0.122	0.014	0.269*	0.108
Bacon	-0.068	-0.085	-0.114	-0.030	-0.087	-0.117
Soy Souce	-0.090	-0.065	-0.115	0.087	-0.061	-0.119
Pickle	0.072	-0.118	0.242	0.159	0.521**	0.232
Sourdough bread	-0.187	-0.127	-0.154	-0.172	0.021	-0.160
Whole grain bread	-0.321*	-0.186	-0.235	-0.164	-0.209	-0.247
Rye bread	0.226	0.143	-0.063	0.186	-0.026	-0.071
Wholemeal bread	0.126	0.095	-0.067	0.013	0.054	-0.071
Ayran	0.131	0.197	0.081	0.069	-0.137	0.080
Kefir	-0.118	-0.149	-0.309*	-0.268*	0.082	-0.320*
Probiotic beverage	-0.114	-0.097	0.151	0.100	-0.077	0.145
Probiotic cheese	-0.147	-0.128	0.165	0.060	-0.100	0.158
Probiotic dark chocolate	-0.171	-0.125	0.036	0.314*	-0.090	0.028
Butter (natural-homemade)	0.121	0.040	0.043	0.029	-0.231	0.048
Probiotic tablet, sachet	-0.068	-0.059	-0.055	0.201	0.128	-0.058
Pomegranate syrup	0.052	-0.048	0.096	-0.097	0.396**	0.091
Vinegar (Homemade)	0.019	-0.077	0.044	-0.030	0.283*	0.037
Others	-	-	-	-	-	-
Gender	-0.147	-0.043	-0.041	0.044	-0.005	-0.055
Age	0.214	0.288^*^	0.275^*^	0.094	0.089	0.283^*^
BMI	0.071	0.168	-0.182	-0.193	0.148	-0.167

*p < 0.05, **p < 0.001

## Discussion

In this study, we tested the hypothesis that regular consumption of fermented foods and probiotic products by individuals with dental implants is positively associated with a lower incidence of peri-implant diseases than individuals who do not consume such foods. The results showed that yogurt, kefir, whole wheat bread, buttermilk, pomegranate syrup, butter (homemade), and vinegar were more consumed in terms of daily intake, general intake, and quantity in peri-implant health patients than in patients with peri-implant diseases.

In the preparation of probiotic yogurt, both ruptured and whole cells of yogurt bacteria (*Lactobacillus delbrueckii ssp. bulgaricus* 2515 and *Streptococcus thermophilus* 2010) are used, along with whole cells of probiotic bacteria (*Lactobacillus acidophilus* 2409 and one species of *Bifidobacterium*; *B. longum* 1941, *B. pseudolongum* 20,099, *B. infantis* 1912, *B. bifidum* 1900 or B. 1901) [30]. These probiotic cultures, including *Lactobacillus acidophilus* and *Bifidobacterium animalis* subsp. lactis, are incorporated into the production process alongside the conventional yogurt bacteria, namely, *Streptococcus thermophilus* and *Lactobacillus delbrueckii subsp. Bulgaricus* [31]. Ayran, which yogurt produced by fermenting milk, is mixed with water and salt. Ayran is industrially produced by adding yogurt cultures to standardized dairy homogenized and pasteurized before fermentation [32]. Bioyogurt probiotics might inhibit bacterial growth of certain species such as *Porphyromonas gingivalis*, *Fusobacterium nucleatum*, *Aggregatibacter actinomycetemcomitans*, *Porphyromonas circumdentaria*, *Prevotella nigrescens*, *P. circumdentaria*, and *Peptostreptococcus anaerobius* [33]. Daily and regular consumption of yogurt containing *Bifidobacterium* microorganisms reduces plaque accumulation and inflammation, and its effect continues as long as it is consumed [34]. The peri-implant health group consumed more yogurt and ayran daily. The peri-implant health group consumed more yogurt per day than the control group.

Kefir is a fermented milk product that contains various strains of the *Lactobacillus* kefir, *Leuconostoc*, *Lactococcus*, and *Acetobacter* genera that are specifically involved in the fermentation process. It is defined as a mixture of *Kluyveromyces marxianus*, which ferments lactose, and nonfermenting yeasts such as *Saccharomyces unisporus*, *Saccharomyces cerevisiae*, and *Saccharomyces exiguus* [35]. Kefir has antibacterial activity against many pathogenic organisms owing to the formation of organic acids (hydrogen peroxide, acetaldehyde, and carbon dioxide) and bacteriocins [36]. Vieira et al. [37] (2021) concluded that milk kefir has anti-inflammatory and anti-resorptive effects on periodontitis in rats, depending on the fermentation time. Patients’ periodontal indices (plaque, gingival index, bleeding on probing, pocket depth, and clinical attachment loss) and the quantity of *T. forsythia* decreased after drinking kefir for 14 days [38]. Regardless of the intragroup decrease in periodontal indices, there was no statistical difference between the test and control groups concerning periodontal indices in this study. The peri-implant health group consumed kefir more generally and daily than the other groups. In addition, unlike the other groups, the peri-implant health group had a higher daily kefir consumption amount.

Polyphenols—phenolic acids, flavonoids, and lignans—are abundant in whole grains [39]. The biological mechanisms of polyphenols, which exhibit anti-inflammatory and antioxidant effects, have been found to mitigate the onset and advancement of periodontitis potentially [40]. The selection of polyphenols at each meal or snack, combined with oral hygiene care measures, prevents periodontitis and other chronic inflammatory conditions that cause it [41]. Nielson et al. [42] (2016) found an inverse relationship between dietary fiber intake and periodontitis among US adults aged < 30 years. In this study, individuals with a healthy peri-implant status consumed whole meal bread daily and generally.

The possible impact of probiotics on peri-implant diseases may be due to their anti-inflammatory effects through their influence on host responses rather than by enhancing the microbial flora in the peri-implant sulcus [36]. Paraprobiotics, which are biotic types like probiotics, are thought to show significant benefits thanks to their immunomodulatory mechanisms of action [37]. After treatment with the *L. reuteri* probiotic tablet, peri-implant mucositis and peri-implant health patients showed indeed improved clinical parameters and reduced cytokine levels [43]. In peri-implantitis patients, probiotic tablets reduced bacterial counts and bleeding on probing [44]. In contrast to the other groups in this study, the peri-implantitis group did not use, however, any probiotic tablets.

It has been reported that apple and grape vinegar may show antibacterial effects against periodontopathogens in vitro (*A. actinomycetemcomitans* and *P. intermedia*) [45]. In this study, the rate of vinegar consumption was lower in the peri-implant health group than in the other groups.

Pomegranate, known for its strong antibiotic, antiviral, antioxidant, anti-inflammatory, wound-healing, and probiotic qualities, could promote periodontal health [46]. When used as an adjunctive measure to subgingival instrumentation, pomegranate extracts in chip or gel form provided additional benefits [47]. In this study, the daily consumption rate of pomegranate syrup was higher in peri-implant health than in peri-implant diseases.

Butter has a unique texture and flavor. Generally, lipids have a desirable impact on the sensory properties of many food products by affecting the mouthfeel, color, texture, and rheological properties. Natural lipids differ in their precise fatty acid content and contain varying amounts of saturated, monoenoic, and polyunsaturated fatty acids [37]. According to Varela-López et al. [48] (2015), the primary dietary methods for achieving periodontal health include replacing saturated fats with monounsaturated fatty acids and polyunsaturated fatty acids (PUFAs), especially n-3 PUFAs. Iwasaki et al. [49] (2011) showed an independent association between dietary saturated fatty acid (SFA) consumption and periodontitis in elderly Japanese nonsmokers.

Despite the novel hypothesis tested in this study, there are several limitations that must be clearly acknowledged. Firstly, the use of a convenience sample may limit the generalizability of the findings. Secondly, there is a possible risk of information bias related to the utilization of questionnaires to assess the consumption of fermentable foods and probiotics. Thirdly, and importantly, potential confounding variables have not been considered, which may influence the study outcomes. This introduces a risk of confounding bias that cannot be ruled out. Therefore, randomized clinical trials are warranted to validate the findings presented in this study. In addition to probiotic studies, future research should explore the effect of parabiotic interventions on peri-implant diseases, especially considering their potential immunomodulatory effects.

## Conclusions

The intake of yogurt, ayran, and kefir, as well as fermented foods such as whole meal bread, whole wheat bread vinegar, pomegranate syrup, and butter (homemade), was associated the peri-implant health among the screened cohort. This study shows that fermented foods and probiotics in the diet, which are natural processes in our lives, may play a protective role for peri-implant diseases.

### Electronic supplementary material

Below is the link to the electronic supplementary material.

Supplementary Material 1

## Data Availability

All data generated or analysed during this study are included in this published article [and its supplementary information files] All data generated or analysed this study are included in this published article [and its supplementary information files].

## Abbreviations

ANOVA: one-way analysis of variance
PUFAs: polyunsaturated fatty acids
SFA: saturated fatty acid
