# Supplementary material for: Fermented foods and probiotic consumption frequency as protective indicators for peri-implant diseases – a cross-sectional study
Source: BMC Oral Health. 2024 Jul 26;24:849. doi: 10.1186/s12903-024-04625-8 (PMC11282801; doi:10.1186/s12903-024-04625-8)
Supplement: Supplementary file 1 — Supplementary Material 1 [file 12903_2024_4625_MOESM1_ESM.docx]

**Table 4:** Comparison of participants' groups by frequency of consumption of foods

|  | | **Every day** | **3-4 per week** | **1-2 per week** | **2 per month** | **1 per month** | **1 per year** | **None** | **p** |
| --- | --- | --- | --- | --- | --- | --- | --- | --- | --- |
| **Yoghurt** | Peri-implantitis | 7 (29,2%) | 8 (33,3%) | 8 (33,3%) | 0 (0,0%) | 1 (4,2%) | 0 (0,0%) | 0 (0,0%) | 0,040* |
|  | Peri-implant mucositis | 5 (25,0%) | 6 (30,0%) | 8 (40,0%) | 0 (0,0%) | 1 (5,0%) | 0 (0,0%) | 0 (0,0%) |  |
|  | Peri-implant health | 12 (75,0%) | 2 (12,5%) | 1 (6,3%) | 0 (0,0%) | 0 (0,0%) | 0 (0,0%) | 1 (6,3%) |  |
| **Probiotic yoghurt** | Peri-implantitis | 0 (0,0%) | 0 (0,0%) | 0 (0,0%) | 0 (0,0%) | 1 (4,2%) | 1 (4,2%) | 22 (91,6%) | 0,496 |
|  | Peri-implant mucositis | 1 (5,0%) | 0 (0,0%) | 0 (0,0%) | 0 (0,0%) | 3 (15,0%) | 1 (5,0%) | 15 (75,0%) |  |
|  | Peri-implant health | 0 (0,0%) | 0 (0,0%) | 0 (0,0%) | 1 (6,3%) | 0 (0,0%) | 1 (6,3%) | 14 (87,4%) |  |
| **Tarhana** | Peri-implantitis | 1 (4,2%) | 4 (16,7%) | 11 (45,9%) | 2 (8,3%) | 5 (20,8%) | 0 (0,0%) | 1 (4,2%) | 0,525 |
|  | Peri-implant mucositis | 2 (10,0%) | 4 (20,0%) | 9 (45,0%) | 1 (5,0%) | 4 (20,0%) | 0 (0,0%) | 0 (0,0%) |  |
|  | Peri-implant health | 4 (25,0%) | 0 (0,0%) | 8 (50,1%) | 0 (0,0%) | 3 (18,8%) | 0 (0,0%) | 1 (6,3%) |  |
| **Sousage** | Peri-implantitis | 0 (0,0%) | 1 (4,2%) | 9 (37,5%) | 5 (20,8%) | 2 (8,3%) | 0 (0,0%) | 7 (29,2%) | 0,561 |
|  | Peri-implant mucositis | 1 (5,0%) | 0 (0,0%) | 9 (45,0%) | 2 (10,0%) | 4 (20,0%) | 1 (5,0%) | 3 (15,0%) |  |
|  | Peri-implant health | 1 (6,3%) | 0 (0,0%) | 5 (31,3%) | 2 (12,6%) | 5 (31,3%) | 0 (0,0%) | 3 (18,8%) |  |
| **Bacon** | Peri-implantitis | 1 (4,2%) | 0 (0,0%) | 0 (0,0%) | 0 (0,0%) | 2 (8,3%) | 2 (8,4%) | 19 (79,2%) | 0,760 |
|  | Peri-implant mucositis | 0 (0,0%) | 1 (5,0%) | 0 (0,0%) | 1 (5,0%) | 3 (15,0%) | 3 (15,0%) | 12 (60,0%) |  |
|  | Peri-implant health | 1 (6,3%) | 1 (6,3%) | 0 (0,0%) | 0 (0,0%) | 1 (6,3%) | 1 (6,3%) | 12 (75,0%) |  |
| **Soy souce** | Peri-implantitis | 0 (0,0%) | 0 (0,0%) | 0 (0,0%) | 0 (0,0%) | 0 (0,0%) | 0 (0,0%) | 24 (100,0%) | 0,221 |
|  | Peri-implant mucositis | 1 (5,0%) | 0 (0,0%) | 0 (0,0%) | 0 (0,0%) | 1 (5,0%) | 0 (0,0%) | 18 (90,0%) |  |
|  | Peri-implant health | 0 (0,0%) | 1 (6,3%) | 0 (0,0%) | 0 (0,0%) | 0 (0,0%) | 2 (12,5%) | 13 (81,3%) |  |
| **Pickle** | Peri-implantitis | 2 (8,3%) | 7 (29,2%) | 10 (43,7%) | 2 (8,3%) | 2 (8,3%) | 0 (0,0%) | 1 (4,2%) | 0,754 |
|  | Peri-implant mucositis | 3 (15,0%) | 4 (20,0%) | 9 (45,0%) | 0 (0,0%) | 1 (5,0%) | 1 (5,0%) | 2 (10,0%) |  |
|  | Peri-implant health | 3 (18,8%) | 4 (25,0%) | 5 (31,3%) | 1 (6,3%) | 0 (0,0%) | 0 (0,0%) | 3 (18,8%) |  |
| **Sourdough bread** | Peri-implantitis | 5 (20,8%) | 5 (20,9%) | 3 (12,5%) | 0 (0,0%) | 1 (4,2%) | 0 (0,0%) | 10 (41,7%) | 0,913 |
|  | Peri-implant mucositis | 8 (40,0%) | 3 (15,0%) | 2 (10,0%) | 0 (0,0%) | 1 (5,0%) | 0 (0,0%) | 6 (30,0%) |  |
|  | Peri-implant health | 5 (31,3%) | 2 (12,5%) | 3 (18,8%) | 0 (0,0%) | 0 (0,0%) | 0 (0,0%) | 6 (37,5%) |  |
| **Whole grain bread** | Peri-implantitis | 4 (16,7%) | 1 (4,2%) | 5 (20,8%) | 0 (0,0%) | 0 (0,0%) | 0 (0,0%) | 14 (58,4%) | 0,577 |
|  | Peri-implant mucositis | 7 (35,0%) | 1 (5,0%) | 2 (10,0%) | 0 (0,0%) | 0 (0,0%) | 0 (0,0%) | 10 (50,0%) |  |
|  | Peri-implant health | 5 (31,3%) | 0 (0,0%) | 2 (12,5%) | 1 (6,3%) | 0 (0,0%) | 1 (6,3%) | 7 (43,8%) |  |
| **Rye bread** | Peri-implantitis | 2 (8,3%) | 2 (8,3%) | 0 (0,0%) | 0 (0,0%) | 1 (4,2%) | 0 (0,0%) | 19 (79,2%) | 0,814 |
|  | Peri-implant mucositis | 3 (15,0%) | 1 (5,0%) | 1 (5,0%) | 0 (0,0%) | 1 (5,0%) | 0 (0,0%) | 14 (70,0%) |  |
|  | Peri-implant health | 2 (12,5%) | 0 (0,0%) | 1 (6,3%) | 0 (0,0%) | 2 (12,5%) | 0 (0,0%) | 11 (68,8%) |  |
| **Wholemeal bread** | Peri-implantitis | 3 (12,5%) | 2 (8,3%) | 1 (4,2%) | 0 (0,0%) | 0 (0,0%) | 0 (0,0%) | 18 (75,0%) | 0,375 |
|  | Peri-implant mucositis | 3 (15,0%) | 3 (15,0%) | 0 (0,0%) | 1 (5,0%) | 0 (0,0%) | 0 (0,0%) | 13 (65,0%) |  |
|  | Peri-implant health | 3 (18,8%) | 1 (6,3%) | 3 (18,8%) | 0 (0,0%) | 0 (0,0%) | 0 (0,0%) | 9 (56,3%) |  |
| **Ayran** | Peri-implantitis | 4 (16,7%) | 5 (20,8%) | 7 (29,2%) | 1 (4,2%) | 5 (20,9%) | 0 (0,0%) | 2 (8,3%) | 0,338 |
|  | Peri-implant mucositis | 4 (20,0%) | 5 (25,0%) | 11 (55,0%) | 0 (0,0%) | 0 (0,0%) | 0 (0,0%) | 0 (0,0%) |  |
|  | Peri-implant health | 5 (31,3%) | 4 (25,0%) | 3 (18,8%) | 0 (0,0%) | 1 (6,3%) | 1 (6,3%) | 2 (12,5%) |  |
| **Kefir** | Peri-implantitis | 1 (4,2%) | 0 (0,0%) | 0 (0,0%) | 3 (12,5%) | 0 (0,0%) | 2 (8,3%) | 18 (75,0%) | 0,032* |
|  | Peri-implant mucositis | 1 (5,0%) | 1 (5,0%) | 2 (10,0%) | 1 (5,0%) | 1 (5,0%) | 1 (5,0%) | 13 (65,0%) |  |
|  | Peri-implant health | 5 (31,3%) | 0 (0,0%) | 2 (12,5%) | 1 (6,3%) | 2 (12,5%) | 0 (0,0%) | 6 (37,5%) |  |
| **Probiotic beverages** | Peri-implantitis | 0 (0,0%) | 0 (0,0%) | 1 (4,2%) | 0 (0,0%) | 0 (0,0%) | 0 (0,0%) | 23 (95,8%) | 0,319 |
|  | Peri-implant mucositis | 0 (0,0%) | 0 (0,0%) | 0 (0,0%) | 0 (0,0%) | 1 (5,0%) | 1 (5,0%) | 18 (90,0%) |  |
|  | Peri-implant health | 1 (6,3%) | 0 (0,0%) | 0 (0,0%) | 0 (0,0%) | 0 (0,0%) | 2 (12,5%) | 13 (81,3%) |  |
| **Probiotic cheese** | Peri-implantitis | 1 (4,2%) | 1 (4,2%) | 0 (0,0%) | 0 (0,0%) | 0 (0,0%) | 0 (0,0%) | 22 (91,7%) | 0,674 |
|  | Peri-implant mucositis | 1 (5,0%) | 1 (5,0%) | 1 (5,0%) | 0 (0,0%) | 0 (0,0%) | 1 (5,0%) | 16 (80,0%) |  |
|  | Peri-implant health | 1 (6,3%) | 0 (0,0%) | 0 (0,0%) | 1 (6,3%) | 0 (0,0%) | 1 (6,3%) | 13 (81,3%) |  |
| **Probiotic dark chocolate** | Peri-implantitis | 0 (0,0%) | 0 (0,0%) | 2 (8,3%) | 0 (0,0%) | 0 (0,0%) | 0 (0,0%) | 22 (91,7%) | 0,068 |
|  | Peri-implant mucositis | 2 (10,0%) | 0 (0,0%) | 0 (0,0%) | 0 (0,0%) | 1 (5,0%) | 1 (5,0%) | 16 (80,0%) |  |
|  | Peri-implant health | 2 (12,5%) | 1 (6,3%) | 1 (6,3%) | 0 (0,0%) | 0 (0,0%) | 1 (6,3%) | 11 (68,8%) |  |
| **Probiotic butter** | Peri-implantitis | 5 (20,8%) | 6 (25,0%) | 2 (8,3%) | 0 (0,0%) | 1 (4,2%) | 0 (0,0%) | 10 (41,7%) | 0,435 |
|  | Peri-implant mucositis | 9 (45,0%) | 2 (10,0%) | 2 (10,0%) | 0 (0,0%) | 0 (0,0%) | 0 (0,0%) | 7 (35,0%) |  |
|  | Peri-implant health | 10 (62,5%) | 0 (0,0%) | 0 (0,0%) | 1 (6,3%) | 0 (0,0%) | 0 (0,0%) | 5 (31,3%) |  |
| **Probiotic tablet, sachet** | Peri-implantitis | 0 (0,0%) | 0 (0,0%) | 0 (0,0%) | 0 (0,0%) | 0 (0,0%) | 0 (0,0%) | 24 (100,0%) | 0,049* |
|  | Peri-implant mucositis | 1 (5,0%) | 0 (0,0%) | 0 (0,0%) | 0 (0,0%) | 0 (0,0%) | 0 (0,0%) | 19 (95,0%) |  |
|  | Peri-implant health | 0 (0,0%) | 0 (0,0%) | 1 (6,3%) | 0 (0,0%) | 0 (0,0%) | 0 (0,0%) | 15 (93,8%) |  |
| **Pomegranate**  **syrup** | Peri-implantitis | 2 (8,3%) | 2 (8,3%) | 1 (4,2%) | 0 (0,0%) | 0 (0,0%) | 3 (12,5%) | 16 (66,7%) | 0,002* |
|  | Peri-implant mucositis | 4 (20,0%) | 0 (0,0%) | 4 (20,0%) | 0 (0,0%) | 2 (10,0%) | 0 (0,0%) | 10 (50,0%) |  |
|  | Peri-implant health | 11 (68,8%) | 0 (0,0%) | 2 (12,5%) | 0 (0,0%) | 2 (12,5%) | 0 (0,0%) | 1 (6,3%) |  |
| **Vinegar**  **(Homemade)** | Peri-implantitis | 6 (25,0%) | 2 (8,3%) | 3 (12,5%) | 0 (0,0%) | 0 (0,0%) | 0 (0,0%) | 13 (54,1%) | 0,041* |
|  | Peri-implant mucositis | 4 (20,0%) | 1 (5,0%) | 6 (30,0%) | 0 (0,0%) | 1 (5,0%) | 0 (0,0%) | 8 (40,0%) |  |
|  | Peri-implant health | 0 (0,0%) | 11 (68,8%) | 1 (6,3%) | 0 (0,0%) | 0 (0,0%) | 1 (6,3%) | 3 (18,8%) |  |
| **Others** | Peri-implantitis | 0 (0,0%) | 0 (0,0%) | 0 (0,0%) | 0 (0,0%) | 0 (0,0%) | 0 (0,0%) | 24 (100,0%) | 0,212 |
|  | Peri-implant mucositis | 0 (0,0%) | 0 (0,0%) | 0 (0,0%) | 0 (0,0%) | 0 (0,0%) | 0 (0,0%) | 20 (100,0%) |  |
|  | Peri-implant health | 0 (0,0%) | 0 (0,0%) | 0 (0,0%) | 0 (0,0%) | 0 (0,0%) | 0 (0,0%) | 16 (100,0%) |  |
